# A generic selection system for improved expression and thermostability of G protein-coupled receptors by directed evolution

**DOI:** 10.1038/srep21294

**Published:** 2016-02-18

**Authors:** Christoph Klenk, Janosch Ehrenmann, Marco Schütz, Andreas Plückthun

**Affiliations:** 1 Department of Biochemistry, University of Zurich, Winterthurerstrasse 190, CH-8057 Zurich, Switzerland

## Abstract

Structural and biophysical studies as well as drug screening approaches on G protein-coupled receptors (GPCRs) have been largely hampered by the poor biophysical properties and low expression yields of this largest class of integral membrane proteins. Thermostabilisation of GPCRs by introduction of stabilising mutations has been a key factor to overcome these limitations. However, labelled ligands with sufficient affinity, which are required for selective binding to the correctly folded receptor, are often not available. Here we describe a novel procedure to improve receptor expression and stability in a generic way, independent of specific ligands, by means of directed evolution in *E. coli*. We have engineered a homogenous fluorescent reporter assay that only detects receptors which are correctly integrated into the inner cell membrane and, thus, discriminates functional from non-functional receptor species. When we combined this method with a directed evolution procedure we obtained highly expressing mutants of the neurotensin receptor 1 with greatly improved thermostability. By this procedure receptors with poor expression and/or low stability, for which no ligands or only ones with poor binding properties are available, can now be generated in quantities allowing detailed structural and biophysical analysis.

By relaying signals from the exterior to the interior of the cell, integral membrane proteins (IMPs) play a central role in the physiological and pathophysiological processes of our body. With more than 800 members, G protein-coupled receptors (GPCRs) represent the largest superfamily of IMPs and are considered as one of the most important drug targets^1^,^2^. However, most GPCRs exhibit several unfavourable features which make them difficult to study. Major reasons for this are, on the one hand, the very low levels of endogenous expression and also the low achievable expression levels in most heterologous systems, resulting in low functional protein yields that can be isolated. On the other hand, owing to their inherent flexibility, most GPCRs are extremely unstable, and instability is further increased after solubilisation of the protein in detergents. Thus, the number of structural studies and studies requiring purified protein of GPCRs has remained limited, compared to the many investigations within the past decades that have employed cell-biological and biochemical methods in whole cells^3^. Likewise, the development of new drugs targeting GPCRs using structure-based drug design or fragment-based drug discovery has remained a challenge^4^, as these methods also require access to purified protein of sufficient stability.

Among other methods, conformational stabilisation by introducing thermostabilising mutations has been a major advancement to improve the biophysical properties of GPCRs. Such modified receptors were the basis for several high resolution crystal structures^5^,^6^,^7^,^8^,^9^, and are well suited for extensive *in vitro* drug screening trials^10^. Stabilising mutations can be found, e.g., by performing successive alanine scans over the whole protein followed by screening for improved receptor stability of each individual mutant. Combinations of several beneficial mutations then lead to highly stabilized receptors^11^,^12^,^13^,^14^,^15^,^16^,^17^. Recently, we have developed a more comprehensive approach to identify such mutations by directed evolution of the receptor^18^, combined with cytometric selection^19^. The overall scheme of the FACS-based selection is shown in Fig. 1. Here, libraries of randomized receptors are expressed in *E. coli* such that functional receptors are integrated into the inner cell membrane. After selective permeabilisation of the outer membrane, fluorescently labelled ligands can bind to the receptor, thus allowing to select the best expressing receptor variants by fluorescence-activated cell sorting (FACS)^18^,^20^,^21^,^22^,^23^. Most of the highly expressing receptor variants obtained by this procedure exhibit improved thermostability^21^,^22^. With a recently developed technology relying on encapsulation of single *E. coli* cells it is even possible to directly select for protein stability in detergent^20^,^24^. Thus, directed evolution of GPCRs not only allows to test millions of receptor variants in a short time but also makes the full amino acid sequence space available to the search for advantageous mutations.

Each of these methods requires the availability of labelled ligands that specifically bind to the receptor of interest with high affinity. While for classical alanine scans typically receptor expression and stability is measured, individually for each mutant, with radioactively labelled ligands^5^,^14^,^15^,^16^,^17^, fluorescently labelled ligands are used for directed evolution to quantify functional receptor levels in the cell^18^,^20^,^21^. However, for many receptors these requirements are not easy to meet. Only for a small proportion of the large family of GPCRs high-affinity ligands with suitable radioactive or fluorescent labels are available, and many ligands exhibit unfavourable features that make them inappropriate for these applications. For the so-called orphan receptors not even a cognate ligand is known, and therefore these receptors have remained inaccessible to conformational stabilisation. But even when a suitable ligand is available, finding the optimal binding condition for each receptor-ligand combination can be laborious and time-consuming, thus preventing the fast processing of many different receptors in parallel, which would be of great interest, especially for drug screening.

The correct folding and integration into the plasma membrane is commonly believed as one of the main bottlenecks for heterologous overexpression of IMPs in *E. coli*^25^,^26^,^27^. Likewise, our previous work suggests that many GPCRs which had been evolved for improved expression and stability exhibit improved biophysical properties, leading to higher folding efficiency and thus to better membrane integration^8^,^18^,^23^. We therefore hypothesized that a direct correlation between receptors that are successfully integrated into the inner cell membrane and functional receptor levels may exist. Thus, quantifying receptor integration in the membrane would give a direct measure for the amount of correctly folded and functional protein in the cell. Based on this hypothesis, we devised a novel method to stabilise GPCRs without the need for a specific labelled ligand using directed evolution in *E. coli*. Our method is fast and should be easily applicable to a wide range of IMPs.

## Results

### Generic detection of functional levels of heterologously expressed GPCRs in *E. coli*

To overcome the boundaries of ligand-dependent selection we searched for an alternative way to discriminate correctly folded, functional from non-functional receptor in *E. coli* that would then allow FACS-based selection for functional expression in a generic way. It has been proposed that a fusion of GFP to the C-terminus can serve as a reporter for correct folding of cytosolic^28^ and also of integral membrane proteins^29^,^30^. It was therefore of interest to test whether such a simple system might also be useful for selecting improved receptors variants from random GPCR libraries. While indeed the stringent selection from a highly diverse library of neurotensin receptor 1 (NTR1)^22^ for high GFP fluorescence leads to a rapid and very strong enrichment of cells with high GFP signals (Supplementary Fig. S1), these were identified as coming from a single deletion mutant, having lost practically the whole GPCR gene, thereby fusing GFP directly behind the promoter. Thus, this rapid and facile enrichment of a false-positive clone uncouples selection for GFP from selection for membrane protein integrity. Importantly, the GPCR library used for these experiments was the same as used for the successful selections described below.

We thus sought for an alternative strategy to quantify intact membrane proteins which would more directly be coupled to the correct membrane insertion. Therefore, we created a system to selectively detect GPCR levels at the inner cell membrane by specifically labelling the extracellular part of the receptor in the periplasmic space with a fluorescent dye. We made use of the Designed Ankyrin Repeat Protein (DARPin) FADA3210 that had been developed recently in our group^31^. FADA3210 binds the weakly fluorescent malachite green derivative MG-2p^32^ with high affinity and enhances its fluorescence more than 10,000-fold upon binding. MG-2p is membrane-impermeable due to its short oxyethylene tail^32^, yet exhibits a molecular weight of only 931 Da, which is well within the limits to be introduced into the periplasmic space by selective permeabilisation of the outer cell membrane^18^,^19^.

**Figure 2:**
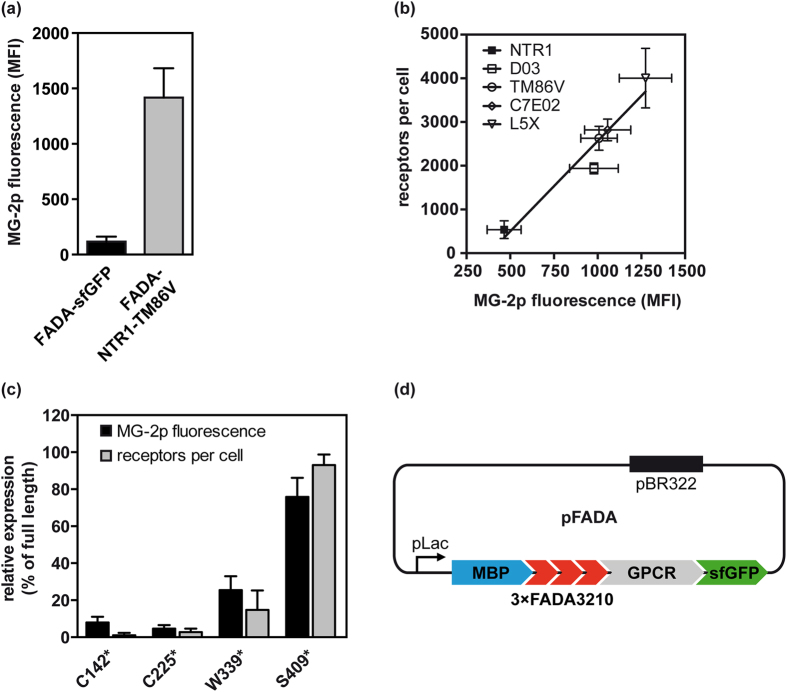
Construction of a generic fluorescent labelling system for periplasmic expression. (**a**) Selective activation of MG-2p fluorescence only in the periplasm. A 3× FADA3210 cassette was expressed in *E. coli* in the cytoplasm as a GFP-fusion protein (FADA-sfGFP), or in the periplasm by anchoring to the N-terminus of NTR1-TM86V (FADA-NTR1-TM86V). Cells were permeabilised with 5× PBS and labelled with MG-2p. Fluorescence activation of MG-2p was measured by flow cytometry and is given as mean fluorescence intensity (MFI) ± S.E.M. of 5 independent experiments. (**b**) MG-2p fluorescence activation correlates with functional receptor expression. 3× FADA3210-fusion proteins of NTR1 and four evolved variants thereof were expressed in *E. coli.* Functional receptor expression was measured by saturating radioligand binding with [^3^H]neurotensin(8–13) and is expressed as receptors per cell. From the same expression cultures, cells were labelled with MG-2p, and fluorescence activation was measured by flow cytometry. The mean fluorescence intensities ± S.E.M. from 5 to 8 independent experiments are given. (**c**) Non-functional receptor fragments are not detected by fluorescence activation. Stop codons were introduced into NTR1-TM86V at positions C^142^, C^225^, W^339^ and S^409^, respectively, and receptor mutants were expressed in *E. coli.* Functional receptor expression was quantified by radioligand binding, and periplasmic fluorescence activation was measured by flow cytometry after labelling with MG-2p. The means, relative to full length NTR1-TM86V expression (+S.E.M.), from 3 independent experiments are shown. (**d**) Schematic representation of the pFADA expression vector used for directed evolution. Maltose binding protein (MBP) including its signal sequence is used for targeting the receptor to the inner cell membrane of *E. coli*^33^, followed by three modules of the fluorescence-activating DARPin FADA3210 (3× FADA3210). sfGFP is fused to the C-terminus as a reporter for frame shifts and stop codons.

To test the feasibility of this approach we measured GPCR expression in *E. coli* with a FADA3210 reporter. As in *E. coli* almost all GPCRs are expressed with fewer than 100 functional copies per cell, we initially used an evolved variant of the rat neurotensin receptor 1 (NTR1-TM86V) as a model, which exhibits high functional expression levels in *E. coli*^22^. To obtain optimal fluorescence activation, a cassette consisting of three FADA3210 modules was fused to the N-terminus of NTR1-TM86V, separated by a short linker. The FADA cassette was preceded by maltose binding protein (MBP) and its signal sequence to target the receptor to the inner cell membrane^18^,^33^. After expression of this construct in the *E. coli* strain DH5α, conditions for optimal permeabilisation of the outer membrane and fluorescence activation were tested using a series of buffers. Highest fluorescence activation and homogeneous labelling of cells were obtained with 5× PBS or PBS-E after 2–4 h labelling time (Supplementary Fig. S2a). In contrast, no fluorescence activation of MG-2p with any of the tested buffers was observed after expression of a control construct (a direct fusion of the sensor to GFP in the cytoplasm; FADA-sfGFP) that is not found in the periplasm (Supplementary Fig. S2b), indicating that MG-2p in turn does not enter the cytoplasm under the chosen conditions. As PBS-E reduced cell viability slightly, 5× PBS was chosen as a labelling buffer henceforth. The apparent affinity of MG-2p binding in the periplasmic space was approx. 120 nM (Supplementary Fig. S2c), thus 1 μM MG-2p was used for subsequent labelling experiments to ensure saturating conditions. With this labelling protocol, an approx. 12-fold increase in fluorescence activation of FADA-NTR1-TM86V compared to background FADA-sfGFP was achieved (Fig. 2a).

To test whether detection of MG-2p fluorescence at the inner cell membrane would allow quantitation of functional receptor levels, wild-type NTR1 as well as 4 previously evolved variants thereof, covering a wide range of functional expression levels^18^,^22^,^33^, were analysed for functional receptor expression and MG-2p fluorescence. As shown in Fig. 2b, a strong correlation between binding of neurotensin and fluorescence activation was observed (R^2^ = 0.94), indicating that the detection of receptors at the inner cell membrane indeed can be used as a measure for functional expression of IMPs.

As the selection system needs to be applicable for directed evolution, we considered that truncated or spliced gene variants, which may occur in the initial libraries, might also be integrated into the inner cell membrane or even be exported into the periplasm together with the FADA-reporter, thus leading to false-positive selection results. We therefore also tested the ability of the FADA-reporter system to discriminate corrupted receptor mutants from intact receptors. For this purpose, stop codons were introduced at various positions of the NTR1-TM86V gene to mimic truncated receptor variants. As expected, mutants with a stop codon proximal to helix 7 of the transmembrane bundle (residue 339) were defective for ligand binding. Importantly, fluorescence activation of these receptor mutants was likewise negligible, suggesting that the MG-2p selection should not be compromised by corrupted receptor mutants (Fig. 2c). This indicates that the short receptor fragments are not efficiently incorporated into the membrane and may be largely degraded. As an additional measure to exclude stop codons or frame shifts within the randomized receptor libraries, superfolder GFP (sfGFP) was fused to the C-terminal end of the receptor in the final expression vector pFADA, thus bracketing the receptor by two independently selectable markers (Fig. 2d).

### Directed evolution of NTR1 by MG-2p fluorescence selection

**Figure 3:**
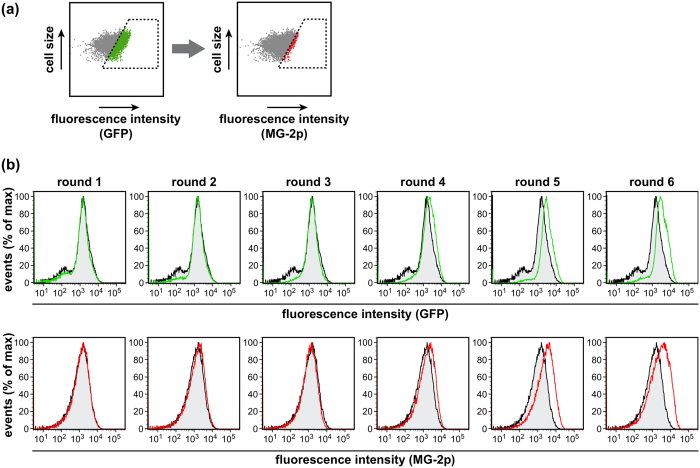
Directed evolution of NTR1 using MG-2p selection. (**a**) Gating scheme for in-frame selection and high expression. A primary sorting gate was set for the top 50% of GFP fluorescent cells. From this population, the top 0.5–1% of MG-2p fluorescent cells were sorted. (**b**) Directed evolution of NTR1 by MG-2p selection. The synthetic library NTR1-D03SLN encompassing approx. 8.5 × 10^8^ variants of NTR1 was cloned into pFADA and expressed in *E. coli*. Cells were labelled with 1 μM MG-2p and sorted as described above in in 6 repetitive rounds. Flow cytometry histogram plots for GFP (upper panels) and MG-2p (lower panels) of selection rounds 1 to 6 (coloured open traces) in comparison to the naïve library (black traces filled in grey) are shown.

The above results encouraged us to apply the labelling system for directed evolution of a GPCR. A highly diverse binary library of the rat NTR1 (based on variant NTR1-D03)^22^ was cloned into the pFADA-reporter construct and expressed in *E. coli*. Cells were labelled with 1 μM MG-2p, and selections were performed by FACS. An initial non-stringent gate was set for GFP-positive cells, merely to exclude mutants with premature stop codons or frame shifts. Thereof, the top 1% of MG-2p fluorescent *E. coli* population was sorted into fresh growth medium, recovered at 28 °C overnight and regrown for five subsequent selection rounds (Fig. 3a). After the first selection round, GFP-negative cells were fully depleted from the pool, and after selection round 3, a marked right-shift of both fluorescence signals was observed. Selections were continued until a stable plateau was reached, constraining the second sorting gate to the 0.5% most fluorescent cells of the MG-2p population during the two final rounds (Fig. 3b). In line with increasing fluorescence, a strong rise in radioligand binding of the selected pool was observed after round 3 which further increased until selection round 6 (data not shown).

**Figure 4:**
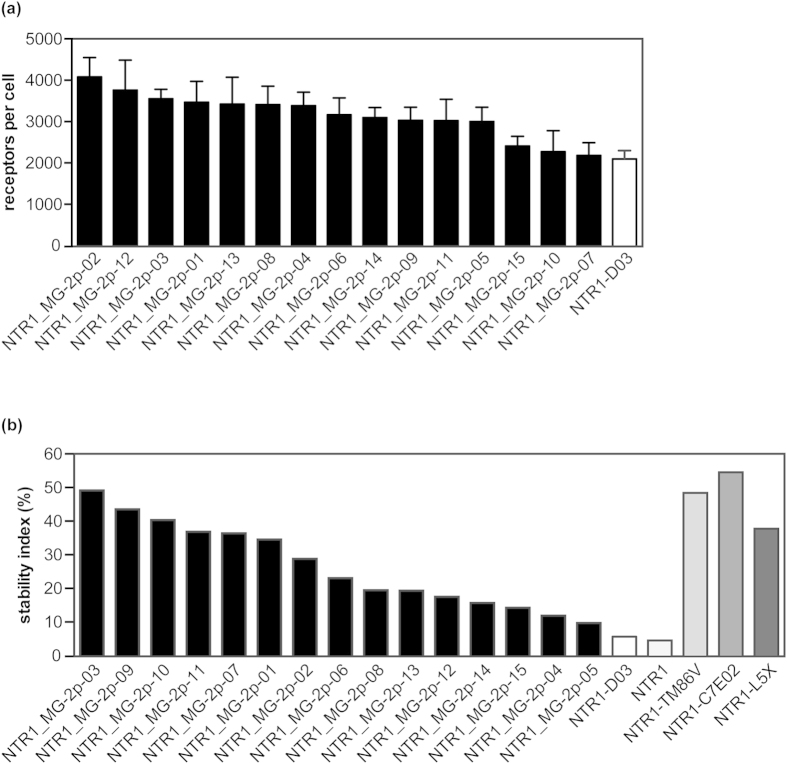
Characterization of evolved NTR1 mutants for expression and thermostability. (**a**) Evolved NTR1 variants exhibit strongly increased expression levels. 15 unique mutants from selection round 6 as well as the underlying variant NTR1-D03 were expressed in *E. coli*, and functional receptor expression was measured by radioligand binding. Means (+S.E.M.) from 6 to 8 independent experiments are shown. (**b**) Evolved NTR1 variants exhibit improved thermostability that is comparable to highly stable NTR1 variants. The thermostability of 15 evolved NTR1 variants from selection round 6, as well as of NTR1-D03 and three highly stable NTR1 variants (TM86V, C7E02 and L5X^22^) was measured after solubilisation in DDM/CHS/CHAPS. The stability index is given as the ratio of remaining ligand binding activity after 20 min of incubation at 60 °C compared to incubation at 4 °C.

From the final selection round, 96 clones were isolated and sequenced. Interestingly, only 15 unique sequences were identified, with the two most frequent sequences accounting for 60% of the complete pool. Each sequence contained between 11 to 21 mutations compared to NTR1-D03 (Supplementary Fig. S3). Based on radioligand binding, each evolved mutant exhibited up to two-fold increased functional receptor expression compared to NTR1-D03 (Fig. 4a). All mutants were also screened for thermostability after solubilisation in a DDM/CHS/CHAPS detergent mix. In comparison to NTR1-D03, each of the selected mutants was more stable after a 20 min heat challenge at 60 °C. Notably, the best variants NTR1_MG-2p-03, −09, −10 and −11 were similarly stable as three of the most stable NTR1 variants (TM86V, C7E02, L5X) that had evolved when fluorescent neurotensin was used for selection^22^ (Fig. 4b). The apparent melting temperatures (*T*_*m*_) of the most stable clones from the MG-2p selections were between 58.1 to 59.4 °C in the absence of ligand, giving an overall increase in *T*_*m*_ of 16 to 17 °C compared to NTR1-D03. In the ligand-bound state, *T*_*m*_ values of 65.1 to 68.0 °C were obtained, which was an increase of 8 to 11 °C over NTR1-D03 (Table 1). Overall, these data are in good agreement with previous experiments where a similar extent of NTR1 thermostabilisation has been achieved^22^.

In conclusion, we present here a generic and versatile system based on a homogeneous fluorescent labelling assay which allows rapid generation and detection of evolved GPCRs in *E. coli* with high expression rates and improved thermostability.

## Discussion

Protein stabilisation through engineering has proven to be a necessity for many biophysical and biochemical studies on GPCRs, foremost for structure determination and drug screening approaches. So far, however, these techniques are cumbersome and only applicable to a rather small set of proteins, as for each receptor suitable ligands have to be at hand and need to be validated. This is true for approaches based on alanine scanning, as well as those on directed evolution.

To overcome these limitations and to facilitate the workflow for receptor stabilisation, we set out to develop a stabilisation method based on directed evolution in *E. coli* which does not rely on labelled ligands. For this purpose, a way had to be found to quantify functional receptors with a fluorescent reporter which would be generically applicable to a variety of different proteins and could be used at the single cell level without compromising cell viability. Notably, a well established approach of using a C-terminal GFP-fusion as a reporter for correct protein folding^29^,^34^ was found to be not suitable for directed evolution. In this case, after only few selection rounds we observed GFP expression to be uncoupled from receptor expression, mostly through deletion events on the plasmid, leading ultimately to the selection for soluble cytoplasmic GFP, which is much brighter than any fusion to an IMP.

From these observations we concluded that a more direct and robust assessment of receptor localization in the membrane was necessary. As a consequence we fused the fluorescence-activating DARPin FADA3210 to the extracellular part of a NTR1 variant, and sfGFP to the C-terminal end. As DARPins are fast folding and highly stable proteins^35^, we reasoned that a N-terminal fusion would not greatly impair receptor expression. In combination with the membrane-impermeable MG-2p fluorogen, which can be introduced exclusively into the periplasmic space through permeabilisation of the outer membrane, selective labelling of receptors embedded in the inner cell membrane in a generic way was possible. This is the key difference to the potential use of an autofluorescent protein on the N-terminus of a GPCR, as FADA3210 in combination with MG-2p will only be fluorescent when located in the periplasm. Moreover, we demonstrate that MG-2p activation directly correlates with functional receptor expression. In contrast to using the C-terminal GFP fusion exclusively, we did not observe uncoupling of MG-2p fluorescence from receptor expression. However, in combination with the FADA3210/MG-2p read-out, the C-terminal GFP fusion was useful to prevent accumulation of stop codons or frameshifts.

Using FADA3210 as fluorescence reporter was a crucial step for successful selection of functional receptor variants. Interestingly, when we used a single-chain antibody engineered for MG-2p fluorescence activation^32^ instead of the DARPin fusion, frameshifts and stop codons accumulated in the selected pools, which led to a complete loss of receptor expression within a few selection rounds. This was caused by proteolytic cleavage at the C-terminus of the antibody fragment, resulting in uncoupling of fluorescent reporter expression and receptor expression. In contrast, FADA3210 fused to several GPCRs showed no or only negligible signs of proteolytic separation (data not shown). Moreover, we found that truncated proteins consisting of only the periplasmic export signal, MBP and the FADA-reporter were largely not detected by MG-2p labelling, thus preventing the selection of incomplete and thus non-functional receptor species. Even though this observation is a critical feature of the selection system, at present we can only speculate on the underlying mechanisms. It is possible that the fast folding nature of DARPins interferes with their export^36^ more in soluble form than when anchored to an IMP.

After six selection rounds, a strong enrichment of highly expressing receptor variants was obtained, and the initially highly diverse library was reduced to only 15 individual mutants that were isolated from the final selection pool. Each of these receptor mutants displayed significantly higher expression rates than NTR1-D03. More importantly, all receptor mutants also exhibited higher thermostability, and the best mutants were equally stable as the best mutants that had been evolved from the same library using fluorescent neurotensin for selection^22^. This is even more remarkable, as in the present case, stabilisation was carried out in the absence of a cognate ligand, yet each of the evolved mutants was still able to bind neurotensin. Even though we cannot exclude that ligand binding properties may have been altered to some extent, we demonstrate here that GPCRs can be conformationally stabilised in the apo-state without loosing their pharmacological properties completely. In line with that, the best mutants exhibited an increase in *T*_*m*_ of more than 15 °C in the absence of ligand (Table 1). Notably, all variants identified in this study differed from previously described NTR1 mutants that had been optimised for improved expression and thermostability by directed evolution or alanine scans^16^,^17^,^22^. However several overlapping key residues were found: L119F and C332V were present in 13 and 11 of the selected variants, respectively, and were also found in clone L5X which had been evolved by directed evolution with fluorescent neurotensin^22^. More strikingly, A86L which had previously been demonstrated to be a critical determinant of receptor stability either by alanine scan^16^,^17^ or by directed evolution^8^,^22^ was also found in 11 of the receptor variants described in this study (Supplemental Fig. S3). In line with previous reports, our data suggest that different combinations of beneficial mutations can likewise improve the properties of a receptor^17^,^23^,^37^.

Such stabilised receptors may therefore be ideal candidates for the determination of GPCR structures in the apo-state which would give important insights into the basic mechanisms of ligand binding and receptor activation, but which has, with the exception of rhodopsin, not been accomplished today. Stabilisation of the apo-state is also pivotal for fragment screening, at least when orthosteric ligands are to be developed. Moreover, as our method can theoretically be applied to any IMP with an extracellular N-terminus, it may not have to be confined to the GPCR superfamily.

## Material and Methods

MG-2p was kindly provided by Alan S. Waggoner (Carnegie Mellon University).

### Plasmid construction

To obtain pFADA, three FADA3210 DNA fragments^31^ were amplified by PCR from a synthetic gene introducing a GGGS linker at the C-terminal end of each DARPin. The resulting fragments were assembled by overlapping PCR and cloned into pRGIII^33^ between MBP and thioredoxin (TrxA) via AgeI and BamHI restriction sites. In addition, TrxA was replaced by sfGFP^38^ carrying a C-terminal Avi-tag using NotI and HindIII restriction sites. A control reporter construct for intracellular expression of the FADA3210 cassette was constructed by fusing three N-terminal FADA3210 modules and C-terminal sfGFP by a short linker sequence. Receptor genes of rNTR1, NTR1-D03, NTR1-TM86V, NTR1-L5X and NTR1-C7E02^18^,^22^ were amplified by PCR and cloned into pFADA between the DARPin cassette and sfGFP using NotI and SpeI restriction sites. Point mutations to generate stop codons in pFADA-NTR1-TM86V were introduced by site directed mutagenesis.

### Growth Conditions

*E. coli* strain ElectroMAX DH5α-E was obtained from Life Technologies. Cells were transformed with the respective plasmids by electroporation and were grown at 30 °C in 2× YT supplemented with 10 mg/mL glucose and 100 μg/mL ampicillin. After reaching saturation, cells were inoculated into fresh 2× YT medium supplemented with 2 mg/mL glucose and 100 μg/mL ampicillin to an OD_600_ of 0.05 and grown at 37 °C. At an OD_600_ of 0.5, cells were induced with 250 μM IPTG, and expression was allowed to proceed at 20 °C for 20 h.

### Membrane permeabilisation and analytical flow cytometry

Cells were collected by centrifugation and washed in ice-cold PBS buffer. Cells were labelled with MG-2p in 1× PBS (137 mM NaCl, 2.7 mM KCl, 8.1 mM Na_2_HPO_4_, 1.8 mM KH_2_PO_4_, pH 7.4), 2.5× PBS, 5× PBS, PBS-E (1× PBS with 1 mM EDTA) or TKCl (50 mM Tris-HCl, 150 mM KCl, pH 7.4). If not stated otherwise, 1 μM MG-2p was used and labelling was carried out for 4 h on ice. For analytical flow cytometry 2 × 10^7 ^cells were labelled and analysed directly in labelling buffer in the presence of MG-2p on a FACSCanto II (BD Biosciences). sfGFP and MG-2p fluorescence were detected in the FITC and APC channels, respectively, and for each measurement, 10^5^ events were recorded. Flow cytometry data were analysed with FlowJo 7.6.5.

### Library generation and sorting

A synthetic binary library based on NTR1-D03 (NTR1-D03SLN) with a theoretical diversity of 8.5 × 10^8^ was used^22^. The library was amplified by PCR, digested with BamHI and SpeI and ligated into pFADA. Ligation products were transformed into electrocompetent DH5α cells (Thermo Fisher). Cells were recovered in 5 ml SOC medium for 1 h at 37 °C and further cultivated in 500 ml LB medium supplemented with 10 mg/ml glucose and 100 μg/ml ampicillin for 12 to 16 h at 28 °C. The final library size was 1 × 10^8^ as estimated from dilution series on agar plates. For library sorting, in each selection round 5 × 10^8^ cells were labelled with 1 μM MG-2p in 5× PBS as described above and sorted on a FACS Aria III automated cell sorter (BD Biosciences) into fresh growth medium. After 12 to 16 h recovery at 28 °C, glycerol stocks were prepared or fresh expression cultures were inoculated.

### Receptor characterization

Radioligand binding experiments were essentially performed as described previously^22^. Briefly, 2 × 10^7^ cells were resuspended in TEBB buffer (50 mM Tris-HCl, 1 mM EDTA, 1 mg/ml bovine serum albumin and 40 μg/ml bacitracin, pH 7.4) containing 20 nM [^3^H]neurotensin(8–13) (PerkinElmer) and incubated for 2 h at 4 °C. Nonspecific binding was determined in the presence of 5 μM unlabelled ligand. Cells were applied to glass fiber filters (Millipore), separated from free ligand using a 96-well vacuum manifold (Millipore) and washed four times with TEBB buffer. Filters were dried for 1 h at 60 °C and allowed to dissolve in OptiPhase Super-Mix (PerkinElmer) for 14 h. Filter-bound radioactivity was measured by liquid scintillation counting (Microbeta 1450 Plus liquid scintillation counter, Wallac).

Stability measurements of evolved receptor variants were essentially performed as described previously^21^. Briefly, receptors expressed in *E. coli* were solubilised in buffer containing 50 mM Tris-HCl (pH 7.4), 200 mM NaCl, 30% (v/v) glycerol, 1 mM EDTA, Complete protease inhibitors (Roche), 40 μg/mL deoxyribonuclease I (Roche), 10 mM MgCl_2_, and detergents (DDM, 1% (w/v); CHAPS, 0.5% (w/v); and CHS, 0.1% (w/v)) and immobilized on Dynabeads MyOne Streptavidin T1 beads (Thermo Fisher). Aliquots of immobilized receptor were then heated to a specific temperature in a PCR thermocycler, washed with solubilisation buffer, and remaining receptor activity was measured by radioligand binding using 20 nM [^3^H]neurotensin(8–13). For stability measurements in the agonist-bound state, receptors were saturated with 150 nM [^3^H]neurotensin(8–13) before the heat challenge. Data were analysed by nonlinear regression fitting using GraphPad Prism 6.

## Additional Information

**How to cite this article**: Klenk, C. *et al*. A generic selection system for improved expression and thermostability of G protein-coupled receptors by directed evolution. *Sci. Rep.* **6**, 21294; doi: 10.1038/srep21294 (2016).

## Supplementary Material

Supplementary Information

## Figures and Tables

**Figure 1:**
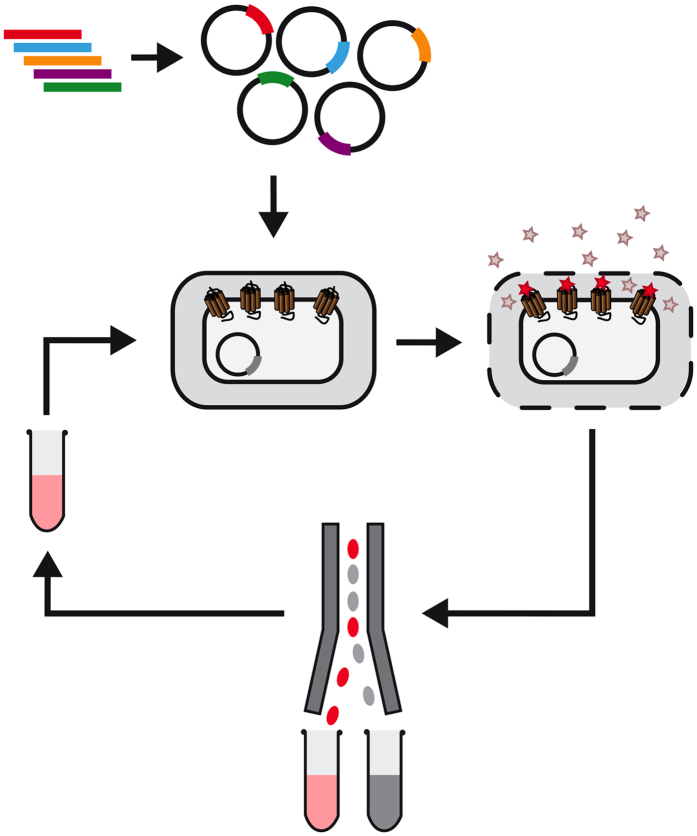
Schematic representation of the directed evolution workflow. First, a random or designed GPCR library is cloned into a suitable expression vector. After expression in *E. coli*, the outer cell membrane is permeabilised and receptors are labelled with fluorescently labelled ligands. Most fluorescent cells are sorted by FACS and recovered in growth medium. To enrich highly expressing receptor variants, repetitive rounds of expression and selection are performed.

**Table 1:** Melting temperatures of the most stable NTR1 variants from the MG-2p selection in comparison to NTR1, NTR1-D03^18^, and NTR1-TM86V^22^.

GPCR	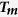 (°C)	
	+NT(8–13)	−NT(8–13)
NTR1_MG-2p-03	65.1 ± 2.3	59.4 ± 2.2
NTR1_MG-2p-09	68.0 ± 3.2	58.2 ± 1.9
NTR1_MG-2p-10	65.8 ± 2.3	59.8 ± 2.5
NTR1_MG-2p-11	66.0 ± 5.7	58.1 ± 1.4
NTR1-TM86V	66.8 ± 3.0	53.3 ± 1.3
NTR1-D03	56.8 ± 0.9	42.5 ± 0.6
NTR1	45.2 ± 0.1	39.2 ± 0.7
