# Supplementary material for: A generic selection system for improved expression and thermostability of G protein-coupled receptors by directed evolution
Source: Sci Rep. 2016 Feb 18;6:21294. doi: 10.1038/srep21294 (PMC4758055; doi:10.1038/srep21294)
Supplement: Supplementary Information [file srep21294-s1.pdf]

Supplementary Information for

A generic selection system for improved expression and thermostability of G protein-coupled receptors by directed evolution.

Christoph Klenk[1], Janosch Ehrenmann[1], Marco Schütz[1,2], and Andreas Plückthun[1]*

[1] Department of Biochemistry, University of Zurich, Winterthurerstrasse 190, CH-8057 Zurich, Switzerland.

[2] present address: G7 Therapeutics AG, Grabenstrasse 11a, CH-8952 Schlieren, Switzerland.

* To whom correspondence should be addressed:

Department of Biochemistry, University of Zurich, Winterthurerstrasse 190, CH-8057 Zurich, Switzerland

Tel.: +41 44 635 55 70

Fax: +41 44 635 57 12

E-mail: plueckthun@bioc.uzh.ch

Supplementary figures

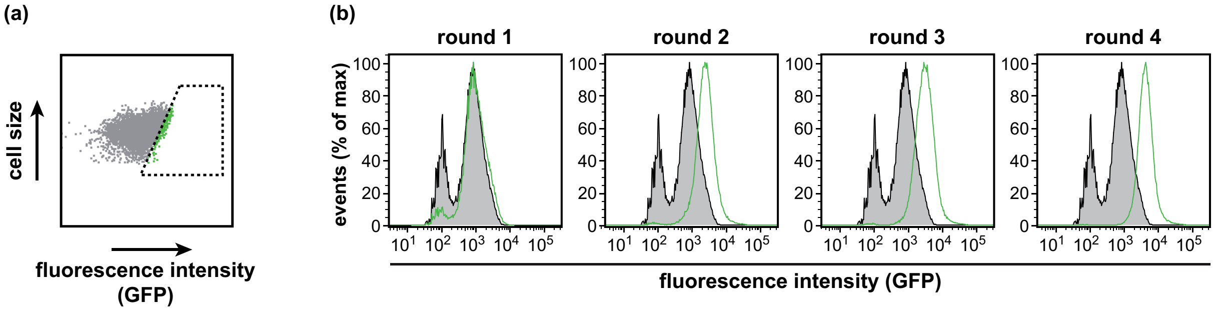

Supplementary Figure S1 | Selections of the NTR1-D03SLN library based only on GFP fluorescence.

To test whether C-terminal fusion of GFP is sufficient to select for highly expressing receptor variants from a randomized library, the synthetic library NTR1-D03SLN encompassing approx. 8.5×10^{8} variants of NTR1 (also used in the other experiments of this study) was expressed with a C-terminal sfGFP fusion in *E. coli* and four rounds of selection by FACS were performed. **(a)** Gating scheme for receptor selection based only on GFP fluorescence. A gate was set to sort the top 1% of GFP-fluorescent cells. **(b)** Flow cytometry histogram plots for GFP of selection rounds 1 to 4 (coloured open traces) in comparison to the naïve library (black traces filled in grey). They show a strong shift of the GFP signal after the first selection round, which further increases during the following rounds. Gating was only for GFP. After selection round 4, ten clones were isolated and analysed by Sanger sequencing. In all isolated clones, the receptor cassette was deleted whereby the GFP reading frame was fused directly to the promoter.

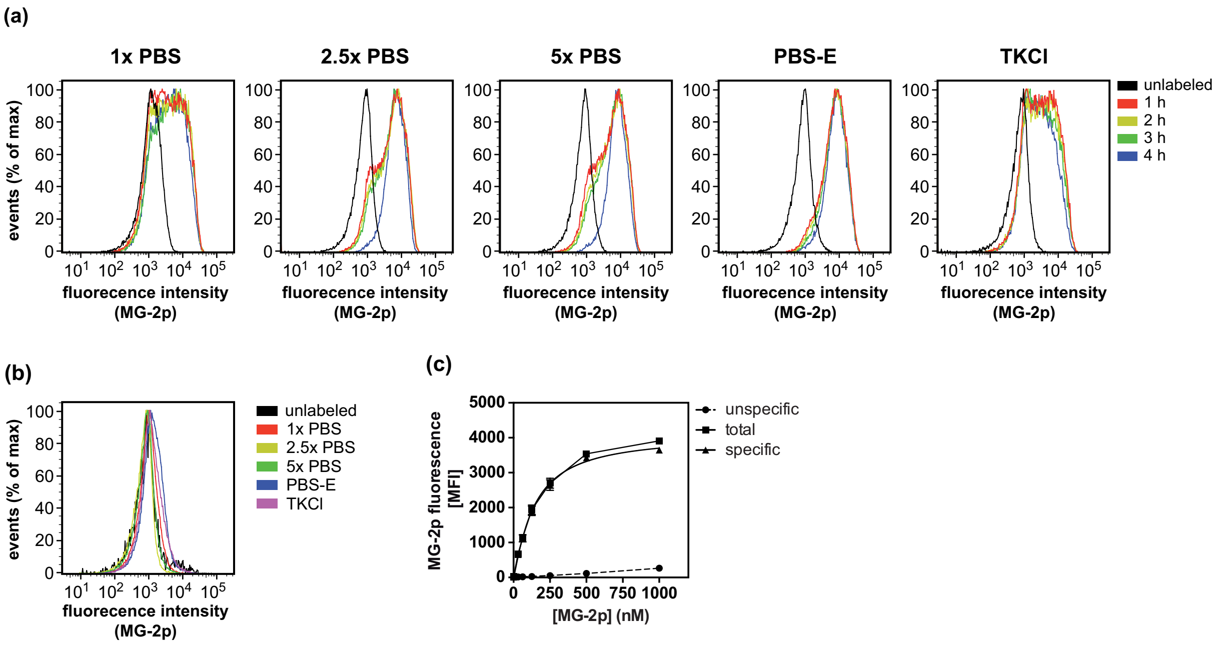

Supplementary Figure S2 | Optimization of MG-2p labelling.

(a) FADA-NTR1-TM86V was expressed in *E. coli*, and 2×10[7] cells were labelled for 1 to 4 h with 1 µM MG-2p in 1× PBS, 2.5× PBS, 5× PBS, PBS-E or TKCl, respectively. MG-2p fluorescence was measured by flow cytometry. Labelling time-dependent MG-2p fluorescence for each buffer is shown as superimposed histogram plots. **(b)** FADA-sfGFP was expressed in *E. coli* and 2×10[7] cells were labelled for 4 h with 1 µM MG-2p in 1× PBS, 2.5× PBS, 5× PBS, PBS-E or TKCl, respectively. MG-2p fluorescence was measured by flow cytometry. MG-2p fluorescence of FADA-sfGFP in each buffer in comparison to unlabelled cells is shown as superimposed histogram plots. **(c)** For saturation binding, *E. coli* cells expressing FADA-NTR1-TM86V were labelled for 4 h with 0 to 1 µM MG-2p in 5× PBS, and unspecific binding was obtained from cells expressing NTR1-TM86V lacking the FADA module. MG-2p fluorescence was measured by flow cytometry and is given as mean fluorescence intensities (MFI) ± S.E.M. of three independent experiments. MG-2p affinity was obtained by a non-linear regression fit, giving an K_d^{app} of 121 ± 2.4 nM.

position	NT		H1					H2							H3				IL2	H4		EL2		H5						H6				H7					
			1.36	1.38	1.43	1.51	1.54	2.38	2.47	2.50	2.56	2.58	2.61	2.62	3.26	3.33	3.49	3.55		4.57	4.58			5.36	5.41	5.54	5.61	5.63	5.64	6.37	6.47	6.51	6.59	7.26	7.38	7.42	7.44	total	sequence
	57	58	68	70	75	83	86	101	110	113	119	121	124	125	143	150	166	172	177	201	202	208	215	235	240	253	260	262	263	310	320	324	332	342	354	358	360	mutations	frequency
**NTR1_D03**	**V**	**N**	**T**	**I**	**F**	**S**	**A**	**T**	**A**	**D**	**L**	**M**	**E**	**L**	**R**	**D**	**E**	**C**	**A**	**A**	**I**	**M**	**G**	**K**	**V**	**I**	**I**	**N**	**K**	**L**	**C**	**Y**	**C**	**F**	**T**	**F**	**V**		
NTR1_MG-2p-01	I	R				G	L		L	S	F		D			E			H			V			L	A		R				L		A	S			17	40%
NTR1_MG-2p-02	I					G	L		L	S	F		D			E			H			V			L	A		R					V					14	20%
NTR1_MG-2p-03	I		S				L			S	F		D	V	K			R	H			V			L				R				V		S	V		16	8%
NTR1_MG-2p-04	I	R			L	G	L			S		L		V	K			R		S	L	V		R		A		R				L	V					18	7%
NTR1_MG-2p-05			S	L	L			R		S	F	L		V	K	E		R	H			V			L			R							S	V		17	5%
NTR1_MG-2p-06	I					G	L		L		F			V		E						V				A	A	R			L	L			S	V		15	4%
NTR1_MG-2p-07	I	R	S		L		L		L		F	L		V	K	E				S	L	V		R	L	A			R				V	A	S			21	4%
NTR1_MG-2p-08	I		S				L			S		L	D	V				R	H							A		R			L			A				13	1%
NTR1_MG-2p-09								R		S	F	L			K				H					R			A						V	A		V		11	1%
NTR1_MG-2p-10	I					G	L		L	S	F		D			E			H			V			L	A		R				H	V					15	1%
NTR1_MG-2p-11		R		L	L	G	L			S	F		D	V	K	E			H			V		R	L	A	A	R				L	V					20	1%
NTR1_MG-2p-12				L		G		R	L	S	F			V	K	E			H	S						A	A		R				V					15	1%
NTR1_MG-2p-13				L	L	G	L		L	S	F	L		V												A					L		V	A	S			14	1%
NTR1_MG-2p-14					L	G	L	R	L		F									S	L			R	L		A		R				V					13	1%
NTR1_MG-2p-15						G		R	L		F									S	L			R	L		A		R		L		V					12	1%
TM86V[1]							L																			A										V		3	
C7E02[1]	I	R	S	L	L	G	L								K	E										A								A	S	V		13	
L5X[1]	I				L			R		S	F	L			K	E			H						L	A					L		V		S	V		15	
NTS1-7m[2]							L																				A							A		A		4	
GW5[3]							L										A						A							A						A	A	6	
Nag36k[3]							L										A													A							A	4	

Supplementary Figure S3 | Comprehensive sequence analysis from MG-2p selection round 6 of the NTR1-D03SLN library in comparison to previously stabilized NTR1 variants.

Mutations of each clone are indicated, and the corresponding wild-type amino acids are given on top of the mutation data. The positions of the mutations are indicated by structural regions, Ballesteros-Weinstein numbering, and sequential amino acid numbering. The frequency of each sequence relative to all sequenced clones is given at the end.

References

1. Schlinkmann, K. M. et al. Maximizing detergent stability and functional expression of a GPCR by exhaustive recombination and evolution. *J. Mol. Biol.* **422**, 414–428 (2012).
2. Shibata, Y. et al. Thermostabilization of the neurotensin receptor NTS1. *J. Mol. Biol.* **390**, 262–277 (2009).
3. Shibata, Y. et al. Optimising the combination of thermostabilising mutations in the neurotensin receptor for structure determination. *Biochim. Biophys. Acta* **1828**, 1293–1301 (2013).
